# Values and Preferences on the Use of Oral Pre-exposure Prophylaxis (PrEP) for HIV Prevention Among Multiple Populations: A Systematic Review of the Literature

**DOI:** 10.1007/s10461-016-1627-z

**Published:** 2016-11-29

**Authors:** Florence M. Koechlin, Virginia A. Fonner, Sarah L. Dalglish, Kevin R. O’Reilly, Rachel Baggaley, Robert M. Grant, Michelle Rodolph, Ioannis Hodges-Mameletzis, Caitlin E. Kennedy

**Affiliations:** 1 Key Populations & Innovative Prevention (KPP), Department of HIV and Global Hepatitis Programme, World Health Organization, 20, Avenue Appia, 1211 Geneva, Switzerland; 2 Department of Psychiatry, Center for Global and Community Health, Medical University of South Carolina, 176 Croghan Spur Rd Suite 104, Charleston, SC 29407 USA; 3 International Health and the Program is Social and Behavioral Interventions, Johns Hopkins Bloomberg School of Public Health, 615 N. Wolfe Street, Baltimore, MD 21205 USA

**Keywords:** Pre-exposure prophylaxis (PrEP), HIV, Tenofovir, HIV prevention, Systematic review, Values and preferences, Multiple populations

## Abstract

Daily oral pre-exposure prophylaxis (PrEP) is the use of antiretroviral drugs by HIV-negative people to prevent HIV infection. WHO released new guidelines in 2015 recommending PrEP for all populations at substantial risk of HIV infection. To prepare these guidelines, we conducted a systematic review of values and preferences among populations that might benefit from PrEP, women, heterosexual men, young women and adolescent girls, female sex workers, serodiscordant couples, transgender people and people who inject drugs, and among healthcare providers who may prescribe PrEP. A comprehensive search strategy reviewed three electronic databases of articles and HIV-related conference abstracts (January 1990–April 2015). Data abstraction used standardised forms to categorise by population groups and relevant themes. Of 3068 citations screened, 76 peer-reviewed articles and 28 conference abstracts were included. Geographic coverage was global. Most studies (N = 78) evaluated hypothetical use of PrEP, while 26 studies included individuals who actually took PrEP or placebo. Awareness of PrEP was low, but once participants were presented with information about PrEP, the majority said they would consider using it. Concerns about safety, side effects, cost and effectiveness were the most frequently cited barriers to use. There was little indication of risk compensation. Healthcare providers would consider prescribing PrEP, but need more information before doing so. Findings from a rapidly expanding evidence base suggest that the majority of populations most likely to benefit from PrEP feel positively towards it. These same populations would benefit from overcoming current implementation challenges with the shortest possible delay.

## Introduction

Oral pre-exposure prophylaxis (PrEP) is the use of antiretroviral drugs (ARVs) by HIV-negative people to prevent HIV infection. In 2010, the first randomized clinical trial results were released showing effectiveness of oral PrEP (tenofovir/emtricitabine) among men who have sex with men [1]. Since then, several more clinical trials have been conducted in different populations, and together these studies suggest PrEP is highly effective if taken regularly [2].

The World Health Organization (WHO) released a first recommendation on the use of PrEP in 2012 [3] for men who have sex with men and serodiscordant couples in the context of demonstration projects to explore strategies for implementation. In 20 [4, 14] WHO recommended offering PrEP for men who have sex with men (MSM) as an additional prevention option. In September 2015, WHO recommended offering PrEP for all persons at substantial risk of HIV infection [5].

In developing evidence-based clinical guidelines, WHO considers the values and preferences of users. For PrEP, as for many other biomedical interventions, understanding the knowledge of potential users, their willingness to use it, and the potential barriers and facilitators to its uptake is critical to its success. Clinical trials demonstrate that adherence to PrEP is significantly associated with effectiveness [2]. Other challenges to the successful implementation of PrEP programs can also be identified and addressed by understanding potential users’ perspectives.

To prepare the 2015 WHO guidance, we conducted a systematic review of the literature on the values and preferences around PrEP across groups that might benefit from it, including heterosexual males, females, and transgender persons, and healthcare providers who may prescribe PrEP.

## Method

### Inclusion Criteria

We included studies in the review if they met the following criteria: (i) examined participants’ views of PrEP, willingness to take PrEP, concerns about PrEP, or values around PrEP, (ii) presented primary data (qualitative or quantitative), and (iii) published as a peer-reviewed journal article or conference abstract. We excluded studies conducted only among men who have sex with men, because a strong WHO recommendation already existed for this population [4]. Think pieces, review articles and consultation reports were not included in the main analysis, and were considered only to verify consistency of findings.

### Search Strategy

We searched PubMed, CINAHL, and EMBASE using the date ranges January 1, 1990 to April 15, 2015. We used the following search terms across all databases: (“pre-exposure prophylaxis” or “preexposure prophylaxis” or “antiretroviral prophylaxis” or “preexposure chemoprophylaxis” or chemoprevention or PrEP) AND (HIV OR AIDS). We also searched abstracts from the following conferences: International AIDS Conference (IAC), Conference on HIV Pathogenesis, Treatment, and Prevention (IAS), and Conference on Retroviruses and Opportunistic Infections (CROI). Only abstracts available electronically were included (CROI 2014–2015, IAS/IAC 2006–2014).

### Data Extraction and Management

All references identified through the search process underwent eligibility screening. Initial screening of database and conference abstract search results was done by one person to remove clearly irrelevant articles. A second screening was then conducted by two reviewers independently with resolution of discrepancies through discussion. Papers meeting the inclusion criteria were obtained in full-text form. All studies were reviewed and key data extracted by a single reviewer using standardized forms. Data included: citation information, population studied, region/country, sample size, key findings, and whether the study was linked to a clinical trial, demonstration project, open label study, family planning clinic, or early roll-out.

We separated findings by the following populations (not mutually exclusive): (1) women, (2) heterosexual men, (3) young women and adolescent girls, (4) female sex workers, (5) serodiscordant couples, (6) transgender people, (7) people who inject drugs, and (8) healthcare providers.

Findings from each study were categorized into five themes to evaluate likelihood of PrEP uptake and factors that may impact uptake and use: (1) awareness of PrEP, (2) willingness to use PrEP, (3) barriers and facilitators to PrEP use, (4) risk compensation, and (5) healthcare providers’ opinions.

## Results

### Description of Included Studies

Appendix Fig 1 presents the disposition of citations through the search and screening process. Of 3068 citations identified through the initial search, 76 peer-reviewed articles and 28 conference abstracts met our inclusion criteria [6–109].

**Fig. 1:**
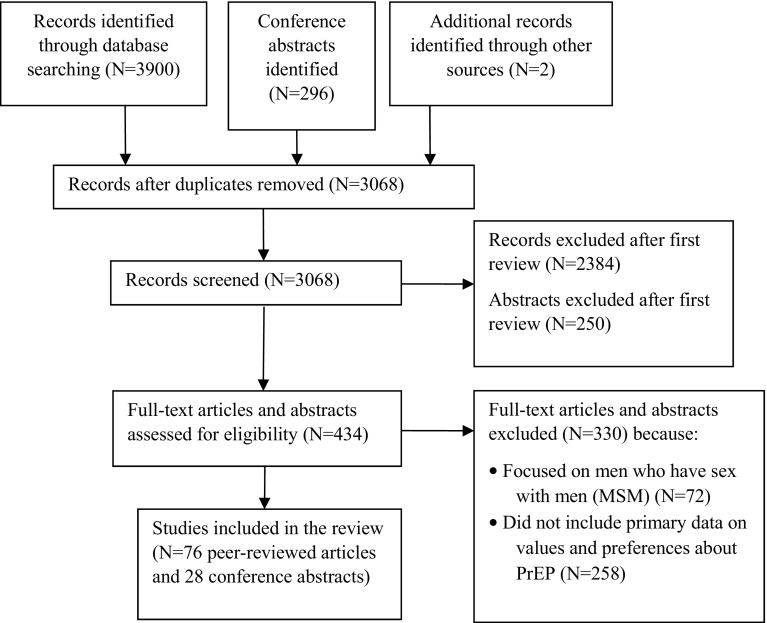
Disposition of citations through the search and screening process

Included studies covered a wide range of population groups and geographic areas (Appendix Table 1). There were more quantitative studies (N = 68) than qualitative (N = 24) or mixed methods (N = 12). Most studies (N = 78) evaluated hypothetical PrEP use or perspectives among people not currently using PrEP, while 26 included people actually taking PrEP or placebo. In this paper, findings reported pertaining to specific studies refer to hypothetical PrEP use unless otherwise specified. In several instances, multiple articles/abstracts appeared to come from the same studies, but without overlapping data. Because it was difficult to clearly identify the number of studies, we refer to articles/abstracts below. In a number of instances, we chose not to list all studies that addressed each point, but picked the ones that best represented the overall themes that emerged, and whenever possible, we selected the larger, higher quality studies, or the studies covering actual use. Note should be made as well that two articles were written in Chinese. The authors based their findings on the English abstract but asked a Chinese speaker to verify that no major inconsistencies existed between the abstract and the body of the Chinese paper.

**Table 1:** Characteristics breakdown

Characteristic	Articles	Abstracts
Location		
Africa: Botswana, Ghana, Kenya, Nigeria, South Africa, Uganda, Zimbabwe	20	13
Asia: China, India, Thailand, Vietnam	16	0
Europe: France, Italy, Switzerland, UK, Ukraine	9	0
Americas: Argentina, Brazil, Canada, Peru, US	38	15
Population		
Women^a^	20	19
Serodiscordant couples	20	6
Female sex workers	11	2
Adolescent girls/young women	5	0
People who inject drugs	5	1
Transgender people^b^	13/7	4/0
Healthcare providers	20	6
Men^c^	11/3	3/0
Study design and method		
Qualitative	20	4
Quantitative	47	21
Mixed method	9	3
Hypothetical use versus actual use		
Hypothetical	59	19
Actual use of PrEP	17	9
Linked to a PrEP clinical trial	11	7
Linked to a PrEP demonstration project/open label study/fertility clinic	6	2
Total	76	28

^a^Not including studies covered in female sex workers, adolescent girls/young women, serodiscordant couples

^b^Study included some transgender people/stratification available or proportion of transgender people high

^c^Not including studies covered in serodiscordant couples. Study included some men/stratification available

### Awareness of and Willingness to Use PrEP

Studies generally reported solid support for PrEP across most populations; however, many studies described a significant lack of knowledge about PrEP and actual use of PrEP remained anecdotal outside of trials. Once the concept of PrEP was introduced, a clear majority of participants across studies welcomed PrEP as a potentially important prevention option for themselves and for others.

#### Women

Among women outside clinical trials or demonstration projects exploring strategies for implementation, awareness of PrEP ranged from ‘almost none’ [7] to 8% having heard about PrEP [102]. One US study mentioned that women were ‘angry’ about not having heard of PrEP [8]. Studies addressing willingness to use PrEP included approximately 15,000 women; willingness to use PrEP ranged from 54% [103] among a group of white Americans to 87% [56] among 5180 women in Kenya. Outside that range, only one study found low interest in PrEP of 20% [43] in a small subgroup of Caribbean women living in the US.

Theoretical interest in PrEP among women was supported by experience from the HPTN 067/ADAPT open-label study. 179 women were given the option to take PrEP, and, knowing it was the active drug, the majority did choose take it [9].

#### Adolescent Girls/Young Women

Just five studies were conducted among adolescent girls and young women. One US study reported that 64% of 595 young women aged 20–29 said they would take PrEP [72]. Another study with Kenyan and South African girls and young women aged 14–24 found that all participants showed strong interest in PrEP [46]. A third study showed participants would be willing to take PrEP if it was provided for free in the US, even if re-testing every 3 months was required [81].

#### Serodiscordant Couples

Studies reported little (3% [55]) to no [76] knowledge of PrEP amongst SDC. Across studies, the majority of serodiscordant couples showed clear willingness to use PrEP, whether to protect seronegative partners or for safer conception. One Chinese study found up to 85% serodiscordant couples were willing to use PrEP [55].

Within the experienced and well-informed Kenyan population of the Partners PrEP clinical trial, one study showed 90% of participants would be willing to use PrEP on a long-term basis against 58% for ‘treatment as prevention’ (TasP), though often preferring the option they controlled [31]. Another Kenyan study, however, found hypothetical TasP to be the more acceptable strategy [23].

Early signs of actual uptake in the general population in the UK were observed in a fertility clinic, where 13 couples offered PrEP chose to take it and 4 declined [100]. Opposite results were seen in South Africa where only 2 out of 16 participants (male and female) elected to take PrEP for safer conception [76].

#### Female Sex Workers

Three Chinese studies reported on PrEP awareness among a total of 2778 female sex workers, with awareness ranging from 12 to 17%. [65, 106, 108] Once the concept of PrEP was introduced, female sex workers across the world expressed strong interest in using PrEP across seven studies (N = 4809 total participants) [18, 24, 46, 65, 68, 106, 108]. Interest rose across these studies over time, in the range of 61–69% in 2010 –2012 [18, 65, 68, 108] up to 86% in 2014 [106].

#### People Who Inject Drugs

Studies indicated that people who inject drugs moderately support PrEP as an additional choice to prevent sexual transmission of HIV. One US study showed 58% of people who inject drugs would use PrEP if it were 90% effective [84]. In Canada, 35% said they would be willing to use PrEP, with higher rates of acceptance among women (42%); younger age, no regular employment, requiring help injecting, engaging in sex work, and reporting multiple recent sexual partners were positively associated with willingness to use PrEP [20]. A small group in a Ukraine study showed higher rates of 86% probable or definite willingness [18].

#### Transgender People

A Thai study reported high PrEP awareness among transgender people (66%); 37% of participants were ‘very likely’ to use PrEP with 50% hypothetical efficacy, increasing to 62% if they had insurance coverage [105]. A majority of transgender people in a small study in the US indicated they would use PrEP [96]. In Peru, transgender people and MSM (results non-stratified) found PrEP ‘highly acceptable’, particularly among those at highest risk [64].

#### Heterosexual Men

We found limited literature on men outside of studies with men who have sex with men, drug users, or serodiscordant couples. One study examined theoretical acceptability of PrEP among truckers and their helpers/cleaners in India with 1602 participants; acceptability of PrEP was 86% [66, 75]. However, a separate paper reported that in-depths interviews with 90 truckers from the same area showed low levels of 33% initial commitment toward PrEP. [74].

### Barriers and Facilitators

The four most commonly cited barriers to PrEP identified in the studies across all risk groups were concerns about safety, side effects, cost and effectiveness. Participants had concerns about safety and whether it could affect their health and well-being, since PrEP involves taking a pill while one is healthy. They were also concerned about potential side effects in the context of other drugs and/or alcohol and/or recreational drugs. Concerns were frequently raised as to how much PrEP would cost, and whether the person using PrEP would have to make a financial contribution. As can be expected, interest in PrEP declines when there is any payment on the part of the user.

Other barriers regularly cited by most risk groups were stigma surrounding HIV and antiretroviral drugs (ARV); low risk perception, including both personal risk and partner risk; the perception that pills are only for sick people; and education level. Facilitators of PrEP use included partner and peer support, especially if peers also knew about PrEP; and discreteness of a pill and the ability to have control over this prevention option, especially in the context of difficulties negotiating condom use.

#### Women

One large US study of 1509 women recorded a higher likelihood of PrEP use among women at high HIV risk with less education, more sexual partners, and provider and peer norms supporting PrEP [103]. The FEM-PrEP clinical trial where PrEP was actually dispensed attributed in part its failure to demonstrate PrEP effectiveness to factors such as unacceptability of a daily pill and negative influence from peers, partners and the community which influenced women not to actually ingest the pills [13]. On the other hand, risk reduction and adherence strategies consisting of external cues, reminders and support facilitated its use [12].

#### Adolescent Girls/Young Women

Young women’s willingness to take PrEP was influenced by their social context. One large US study found young women aged 20–29 were likely to experience stronger social influences (healthcare providers’ recommendation to take PrEP or belief peers would take PrEP) on PrEP uptake than older women, and 77% expected they could adhere to a daily regimen [72]. Another study described young women’s concerns about the difficulty negotiating PrEP use with their partners, and adolescent girls and young women appreciated the ‘privacy’ of a pill [46].

#### Serodiscordant Couples

A Kenyan study found a significant concern among serodiscordant couples that ARVs should not be taken by HIV-negative people, while HIV-positive individuals felt guilty that their HIV-negative partners had to take ARVs because of their own infection [23]. Opinions about the effect of stigma on PrEP use were mixed [23, 55]. Partner support was also considered important in the Partners PrEP study where participants took PrEP, where it was seen as ‘preserving’ the relationship [95]. On the other hand, in the VOICE trial the lack of men’s acceptance of PrEP pertained to men’s unwillingness to accept potential shifts in their relationship power, and may have negatively affected women’s adherence to PrEP [58].

#### Female Sex Workers

In a Phase I study in Kenya with actual PrEP use, female sex workers perceived it positively as a female-controlled HIV prevention option, though they saw incompatibilities between regular pill taking and irregular lifestyles and feared being perceived as HIV positive [90]. One study called for effective follow-up systems to support adherence to clinic and testing visits, as well as schedule cards, home visits and calls [47]. High alcohol consumption by some sex workers was also a repeated concern [46]. Three Chinese studies found that the higher a sex worker assessed her own HIV risk, the more likely she was to find PrEP acceptable [68, 106, 108].

#### People who Inject Drugs

People who inject drugs considered cost and daily dosing requirements as potential barriers to uptake. Blood tests, continued condom use, clinician visits, and regular HIV tests were seen as smaller barriers [84].

#### Transgender People

Cost affected willingness to use PrEP among transgender people in two studies in Peru, [24] where out-of-pocket cost had the greatest impact on decision making, followed by effectiveness, side effects, dispensing location and person [15]. Concerns were expressed about rejection, discrimination and lack of sensitivity from healthcare providers dispensing PrEP [24, 62]. Nearly three quarters of Thai respondents were concerned about drug interaction with hormone replacement therapy and other medications [105].

### Risk Compensation

Many studies considered the potential for risk compensation through increased number of partners and decreased condom use. Across studies, the majority of participants did not anticipate hypothetical PrEP use would lead to increased risk behaviours. These findings are consistent with results from a meta-analysis of PrEP outcomes, which showed no significant effect on sexual behaviour with PrEP use [2]. Some specific groups, however, showed differing results as described below.

#### Women

In one US study, 26% of 1543 respondents indicated that taking PrEP could result in a decrease in condom use [103]. On the other hand, within the small double blinded clinical trial of 400 women in Ghana where PrEP was actually taken, results showed no increase in risk behaviour overall, and a decrease in number of sexual partners and rate of unprotected sex acts [30].

#### Serodiscordant Couples

Just 3% of Chinese participants expected they would increase their number of partners if they were taking PrEP, while 12% reported they would decrease condom use [55]. In Kenya, 25% of respondents indicated a desire to stop using condoms if taking PrEP [23].

#### Female Sex Workers

Some sex workers raised concerns that their colleagues might see PrEP as an opportunity to forego condoms to increase earnings [46].

#### Adolescent Girls/Young Women

One study found 20% of young women expected to use condoms less frequently if they took PrEP [72].

#### Transgender People

One study found anecdotally that condom use may decrease with PrEP [24].

### Healthcare Providers

Across 20 studies and 6 abstracts, providers reported on their knowledge of PrEP. An increase in PrEP awareness over time was observed [101]. It increased up to 90% familiarity with PrEP in 2013 [87] in a survey amongst HIV specialists and HIV healthcare providers after the iPrEx study results became available. Despite this increase, providers across the globe remain reluctant to provide PrEP. Between 9% [35, 36] and 19% [87] of clinicians had prescribed PrEP and 22% of pharmacists [78] had dispensed it. Two studies showed that the likelihood of prescribing PrEP increased when providers cared for more HIV-positive patients, [35] had higher PrEP knowledge, were older, and believed PrEP would empower women.

Most healthcare providers considered PrEP primarily for serodiscordant couples, although some recognised that other groups would benefit. In Italy, 70% of HIV specialists said they would prescribe PrEP, 64% to serodiscordant couples but also 56% to other people at risk, [67] while in Argentina, 40% would consider prescribing PrEP to serodiscordant couples and 35% to sex workers [82]. Medical and counselling service providers supported PrEP in outpatient drug treatment clinics [83].

A common theme across studies was that knowledge of and demand for PrEP should be increased through providing information to healthcare providers, [79] community education campaigns, [6] normative guidance, [79] and local implementation guidelines [85].

## Discussion

Our review identified strong interest and support for PrEP use amongst most populations at risk of HIV infection, and lesser (though still fairly high) interest among people who inject drugs. Notably, literature on heterosexual men, transgender people, adolescent girls and young women, and people who inject drugs is limited, thus calling for more research. Positive responses found in studies evaluating hypothetical PrEP use were supported by studies examining actual use and uptake. Nonetheless, many potential PrEP users and healthcare providers lack basic knowledge of PrEP. Additional efforts are needed to raise awareness about PrEP benefits and ensure more accurate HIV risk perception, especially among younger populations.

The four key barriers to PrEP uptake identified in this review—safety, side effects, cost and effectiveness—can all be addressed through strong programs. Evidence regarding the safety and effectiveness of PrEP is mounting, most side effects associated with oral PrEP diminish after the first month, and costs should drop with increased PrEP availability and insurance coverage. Population- and setting-specific solutions could address other barriers and leverage facilitators to PrEP. For example, late-night clinics may help reach sex workers with irregular schedules [47]. Concerns about potential drug interaction with hormone replacement therapy should be amenable to information campaigns. While some respondents found daily dosing ‘incompatible with a lifestyle where most people ‘live in the moment,’ [24] one study found that over 50% of transgender people were using other daily medications, allowing integration of PrEP into their routine [105]. Further research is however needed to evaluate what best solutions may be to tackle each of these challenges.

Findings from this review suggest limited concern around risk compensation. This is supported by behavioural and reproductive health outcomes from PrEP clinical trials [2] and emerging ethnographic research within the iPrEx open label study (OLE) and the San Francisco PrEP demonstration project that suggests actual use of PrEP may encourage sexual mindfulness and lead to safer behaviour [110, 111]. However, risk compensation may be a concern for specific sub-populations, such as young women, transgender people and sex workers. Those who are highly motivated and adhere to PrEP may not require additional HIV prevention strategies, but may still need protection against pregnancy or other sexually transmitted infections.

Although potential PrEP users expressed interest and enthusiasm about PrEP in the studies considered in this paper, concerns around PrEP undermining existing successful prevention programmes for sex workers were noted by international panels of advocates in settings where comprehensive condom programing is well established and HIV incidence is low [112, 113]. Similar concerns have been raised in using PrEP among people who inject drugs to prevent parenteral HIV transmission, as harm reduction programmes are highly successful in preventing HIV transmission and have broader health benefits [114, 115].

Findings from this review must be seen in light of its limitations. Given a short timeline to provide results for the WHO guideline development process, only one person extracted data. Although we consider our search to be comprehensive, we may still have missed some relevant articles. We note that the current evidence base includes more articles about hypothetical PrEP use than about actual experiences with PrEP. However, as PrEP use expands globally, more studies will likely report actual users’ experiences. We encourage future researchers to consider the gaps identified through this review as opportunities for future research into PrEP values and preferences among diverse populations and in diverse implementation settings.

## Conclusions

PrEP is increasingly recognised as a critical component of comprehensive HIV prevention programming. This systematic literature review on values and preferences confirms that many potential users are interested in PrEP and willing to take it. While multiple implementation challenges remain for countries as they consider the introduction of oral PrEP as an HIV prevention tool, a clearer understanding of how potential users perceive PrEP will enhance the service delivery of PrEP across countries. At risk populations will greatly benefit from overcoming these programming issues with the shortest possible delays.

## Appendix

See Fig 1 and Table 1.
